# Carbonyl reductase 1: a novel regulator of blood pressure in Down syndrome

**DOI:** 10.1042/BSR20241636

**Published:** 2025-02-26

**Authors:** Alexandra J. Malbon, Alicja Czopek, Andrew M. Beekman, Zoë R. Goddard, Aileen Boyle, Jessica R. Ivy, Kevin Stewart, Scott G. Denham, Joanna P. Simpson, Natalie Z. Homer, Brian R. Walker, Neeraj Dhaun, Matthew A. Bailey, Ruth A. Morgan

**Affiliations:** 1 The Royal (Dick) School of Veterinary Studies, The University of Edinburgh, Easter Bush Campus, EH25 9RG, U.K.; 2 The Roslin Institute, The University of Edinburgh, Easter Bush Campus, EH25 9RG, U.K.; 3 Centre for Cardiovascular Science, The Queen’s Medical Research Institute, The University of Edinburgh, Edinburgh, EH16 4TJ, U.K.; 4 School of Pharmacy, University of East Anglia, Norwich Research Park, Norwich, Norfolk, NR4 7TJ, U.K.; 5 Department of Animal and Veterinary Sciences, Scotland’s Rural College, Roslin Institute Building, Easter Bush Campus, EH25 9RG, U.K.; 6 Mass Spectrometry Core, Edinburgh Clinical Research Facility, Queen’s Medical Research Institute, University of Edinburgh, EH16 4TJ, U.K.; 7 Clinical and Translational Research Institute, Newcastle University, Newcastle upon Tyne, U.K.

**Keywords:** blood pressure, carbonyl reductase 1, down syndrome, sympathetic drive

## Abstract

Approximately one in every 800 children is born with the severe aneuploid condition of Down syndrome (DS), a trisomy of chromosome 21. Low blood pressure (hypotension) is a common condition associated with DS and can have a significant impact on exercise tolerance and quality of life. Little is known about the factors driving this hypotensive phenotype, therefore therapeutic interventions are limited. Carbonyl reductase 1 (CBR1) is an enzyme contributing to the metabolism of prostaglandins, glucocorticoids, reactive oxygen species and neurotransmitters, encoded by a gene (*CBR1*) positioned on chromosome 21 with the potential to affect blood pressure. Utilising telemetric blood pressure measurement of genetically modified mice, we tested the hypothesis that CBR1 influences blood pressure and that its overexpression contributes to hypotension in DS by evaluating possible contributing mechanisms *in vitro*. In a mouse model of DS (Ts65Dn), which exhibits hypotension, CBR1 activity was increased and pharmacological inhibition of CBR1 ed to increased blood pressure. Mice heterozygous null for *Cbr1* had reduced CBR1 enzyme activity and elevated blood pressure. Further experiments indicate that the underlying mechanisms include alterations in both sympathetic tone and prostaglandin metabolism. We conclude that CBR1 activity contributes to blood pressure homeostasis and inhibition of CBR1 may present a novel therapeutic opportunity to correct symptomatic hypotension in DS.

## Introduction

Down syndrome (DS) is the most common chromosomal disorder, affecting approximately 1 in every 800 babies born [1]. Ninety-five per cent of people with DS have a trisomy of chromosome 21 with resultant effects on development. Patients with DS are at risk of comorbidities including hypothyroidism, sleep apnoea, obesity, metabolic syndrome, psychiatric disorders and Alzheimer’s disease [2,3]. Low blood pressure – hypotension – is common in both children and adults with DS [4–6]. This hypotension results in lower cardiorespiratory fitness and an inadequate blood pressure response to sub-maximal and maximal exercise [7,8], limiting the ability to participate in many activities [9] which, in turn, affects the quality of life. DS patients also commonly have non-dipping nocturnal blood pressure and heart rate which may contribute to sleep disorders and an increased risk of cardiovascular events [10–12]. There is additionally a well-documented association between low blood pressure and the development of Alzheimer’s disease which is particularly common in patients with DS [5]. This baseline hypotension makes the interpretation of blood pressure as a diagnostic tool for detecting other co-morbidities challenging [4]. Despite these impacts, the pathogenesis of hypotension in DS has not been elucidated; some have suggested that it is due to autonomic dysfunction since clinical studies report reduced sympathetic and increased parasympathetic tone in patients with DS [13–17].

*CBR1,* the gene encoding the ubiquitously expressed enzyme carbonyl reductase 1 (CBR1) [18], is located in the ‘Down syndrome critical region’ of chromosome 21, the region that co-segregates with many of the developmental features of DS [19,20]. CBR1 is a complex enzyme with a number of substrates and is most often studied for its role in metabolism of therapeutics such as doxorubicin [21]. CBR1 is found in almost every cell including the vasculature (endothelial and smooth muscle cells), the heart, liver, kidney and throughout the brain [22,23] (*Tissue Cell Type - IGHG1 - The Human Protein Atlas*). CBR1 plays a critical role in cellular homeostasis and blood flow regulation by preventing the accumulation of reactive oxygen species, vasoconstrictor prostaglandin E_2_ (PGE_2_) and neuroactive metabolites such as monoamine oxidase inhibitors and endogenous indoles [22–27]. Recent data suggest that CBR1 activity is important in regulating renal blood flow via prostaglandin metabolism [28]. Our work has also shown the role of CBR1 in tissue metabolism of glucocorticoids [29] and its impact on glucose homeostasis in lean mice [30]. In this study, we used a transgenic murine model of *Cbr1* deletion, as well as pharmacological inhibition of CBR1 in a murine model of DS, to address the hypothesis that CBR1/*Cbr1* plays a role in blood pressure regulation and that dysregulation of CBR1 contributes to hypotension in DS. We also explore the potential mechanisms by which this might occur.

## Materials and methods

### Animals

All experiments were performed at the Queen’s Medical Research Institute, University of Edinburgh in accordance with the UK’s Animals (Scientific Procedures) Act under a UK Home Office Project Licence in accordance with EU Directive 2010/63/EU. Male B6EiC3Sn.BLiA-Ts(1716)65Dn/DnJ (Ts65Dn), a model of DS, with littermate controls were obtained from The Jackson laboratory (RRID:IMSR_JAX:005252) [31]. This line contains a partial trisomy encompassing most of the human chromosome 21 orthologous region of mouse chromosome 16 [32], including *Cbr1 [33]*. These animals have been well characterised with regard to cerebellar volume which is reduced, as in DS [31]. They demonstrate increased locomotor activity and energy expenditure [34], have reduced blood pressure [35] and impaired conscious respiration associated with a decreased neural drive [36]. Mice heterozygous for *Cbr1 (Cbr1^+/^*^−^) were generated as previously described [30], and homozygosity of this gene deletion is foetal lethal [37]. Data from our group have previously shown that this model has an approximately 50% reduction in CBR1 expression and activity [30]. Mice were maintained according to institutional guidelines, group housed at 21 ± 1°C; humidity at 50 ± 10% with a 12-hour light–dark cycle (light period 07:00–19:00) unless otherwise stated. Mice were randomly allocated to cage, and all environmental factors were kept the same between cages to minimise bias. Unless otherwise specified, mice were killed by cervical dislocation. Mice were fed on a diet containing 0.3% Na and 0.7% K by weight (RM1 diet, Special Diet Services, U.K) throughout the experiment unless otherwise stated. None of the mice included at the start of the study were excluded from any analysis. Blood pressure was measured by telemetry in *Cbr1^+/^*^−^and *Cbr1^+/+^* littermate controls at baseline and during a high-salt diet, and in Ts65Dn mice and their littermate controls at baseline and during treatment with hydroxy-PP-Me, an inhibitor of CBR1 [38,39]. Hydroxy-PP-Me was synthesised using modifications of methods previously described [39]. Renal function, vascular function, plasma renin, angiotensin and aldosterone were measured in mice heterozygous for *Cbr1* and their littermate controls.

### Blood pressure measurement

Ten-week-old male mice (*Cbr1^+/−^, Cbr1^+/+^*, Ts65Dn mice and wild-type littermates (*n* = 8/group)) had PA-C10 radio-telemetry devices (Data Science International, U.S.A.) implanted into the carotid artery under isoflurane anaesthetic (4% induction, 2–3% maintenance). Buprenorphine (0.1 mg/kg Vetergesic; Ceva Animal Health Ltd, Libourne, France) was administered subcutaneously prior to recovery and per os (Vetergesic jelly) for the first four days. Mice were randomly assigned to the order of surgery. Mice underwent a one-week post-surgical recovery period as basal diurnal rhythmicity of the measures was re-established. Data were obtained for the following seven days. For the duration of the experiment, five consecutive 1-minute blood pressure and heart rate readings were taken every 30 minute at an acquisition rate of 1 kHz.

Ts65Dn mice and their wild-type controls then received hydroxy-PP-Me for 1 week during which data were collected. Hydroxy-PP-Me was administered intraperitoneally at a dose of 30 mg/kg based on previously published data [39]. Previous work from our group showed that there was no effect of intraperitoneal injection alone on blood pressure [40]. *Cbr1^+/^*^−^ and *Cbr1^+/+^* littermates did not receive the CBR1 inhibitor but did receive a high-salt diet (3% Na) for seven days (see supplementary data). Raw data are available on request from the authors.

### CBR1 activity

CBR1 activity, as measured by reduction in the substrate doxorubicin, was quantified in hepatic, brain or cardiac cytosol from Ts65DN animals and their littermate controls with or without administration of hydroxy-PP-Me (*n* = 6/group), as previously described [41–43]. Briefly, cytosol from homogenised tissue was extracted by ultracentrifugation, the protein quantified by Bradford protein assay. Cytosol was incubated with 50 µM doxorubicin, and the reaction was started by addition of cofactor NADPH whose oxidation was measured at 340 nm at 37°C over 3 minutes. Enzymatic velocities were calculated by linear regression of the change in absorbance over time.

### Urine collection and analysis

For collection of urine, mice were housed in metabolic cages for 48 hours (*n* = 8–10/group). Urinary catecholamines adrenaline and noradrenaline were measured by enzyme-linked immunoassay (ELISA) (CatCombi ELISA Kit, Creative Diagnostics, DEIA1663). PGE_2_ metabolite was measured by ELISA (Cayman Chemical, 514531) according to the manufacturer’s protocol. Urinary 8-hydroxy-2’-deoxyguanosine (8-OHdG) was measured by ELISA (Abcam, ab201734) according to manufacturer’s instructions.

### Quantitative qPCR

The liver, heart and kidneys from mice were harvested and snap frozen in liquid nitrogen at post-mortem. One kidney from each animal was separated into cortex and medulla prior to freezing. RNA was isolated using RNeasy kits (Qiagen, U.S.A.) and quantified using spectrophotometry (NanoDrop-1000, Thermo Fisher Scientific, U.K), and 500 ng cDNA was synthesised using high-capacity RNA-to-cDNA kit (Thermo Fisher Scientific, U.K). mRNA abundance of relevant transcripts was measured by quantitative RT-PCR using the Universal Probe Library (Roche, U.K). Triplicates of each sample and standard curve were run on the LightCycler 480 (Roche, U.K). Expression was normalised to the mean concentration of housekeeping genes (Table 1).

**Table 1:** Details of primers used in qPCR

Gene symbol, full name	Accession number			Tm	Product length
18S ribosomal RNA (Rn18s)	NR_003278.3	Forward primer (3′→ 5′)	GTAACCCGTTGAACCCCATT	58.09	151
		Reverse primer (5′→ 3′)	CCATCCAATCGGTAGTAGCG	57.93	
Cbr1, carbonyl reductase 1	NM_007620.3	Forward primer (3′→ 5′)	CCCGAGATGTCTGCAAGGAG	60.18	142
		Reverse primer (5′→ 3′)	TCTGTGATGGTCTCGCTTCG	59.83	

### Renal function and salt handling

*Cbr1*^+/−^ and wildtype littermates (*n* = 6/group) were anaesthetised (thiobutabarbital; Inactin; Sigma-Aldrich, Darmstadt, Germany; 120 mg/kg intraperitoneally), the jugular vein cannulated, and isotonic saline containing 0.25% fluorescein isothiocyanate-inulin (FITC-inulin) infused. The carotid artery was cannulated for blood sampling and measurement of BP (Powerlab, AD Instruments, U.K). Following baseline measurements, hydrochlorothiazide was injected intravenously (2 mg/kg hydrochlorothiazide in 0.9% NaCl and 1% DMSO) [44]. Arterial blood was sampled every 40 minutes on three occasions, separated using Haematospin 1400 (Hawksley, U.K) and haematocrit read using Microhaematocrit Reader (Hawksley, U.K). FITC-Inulin was measured by fluorescence (Tecan Sunrise, Tecan Lifesciences, Switzerland) in urine and arterial samples for calculation of glomerular filtration rate.

### Histological examination

Following perfusion fixation, kidneys were collected from 8-week-old male *Cbr1*^+/−^ mice and *Cbr1*^+/+^ littermates (*n* = 4/group). These were longitudinally sectioned and routinely processed through graded alcohol into paraffin prior to sectioning at 2 µm and staining with haematoxylin and eosin. The sections were examined by a board-certified veterinary pathologist.

### Vascular function

Eight-week-old male *Cbr1^+/^*^−^ and wildtype littermates (*n* = 6/group) fed a control diet (0.3% Na) were subject to cervical dislocation after which second-order mesenteric arteries were immediately harvested, submerged in physiological salt solution (PSS; mM: 119.0 NaCl, 4.7 KCl, 2.5 CaCl_2_, 1.17 MgSO_4_, 25.0 NaHCO_3_, 1.81 EDTA, 5.5 D-glucose) and cleaned of adherent perivascular adipose tissue. Wire myography (DMT, Denmark) was used to evaluate the reactivity of the vessels. Vessels were equilibrated under passive tension. Vessel viability was assessed using consecutive stimulations with high potassium physiological saline solution (KPSS, 125 mM) followed by a washout period. Cumulative concentration–response curves were obtained for vasoconstrictors phenylephrine (1 × 10^-9^ – 1 × 10^-4^ M), noradrenaline (1 × 10^-9^ – 1 × 10^-4^ M), 5-hydroxytryptamine (5HT) (1 × 10^-9^ – 1 × 10^-4^ M) and endothelin 1 (1 × 10^-12^ – 1 × 10^-6^ M). Following contraction with phenylephrine to produce 80% of the KPSS response, a cumulative concentration–response curve was obtained for acetylcholine (1 × 10^-9^ – 1 × 10^-4^ M) and sodium nitroprusside (1 × 10^-9^ – 1 × 10^-4^ M).

### Markers of oxidative stress

Plasma was collected from animals at cull. Brains were harvested at post-mortem, snap frozen in liquid nitrogen and stored at −80°C. Total antioxidant capacity was measured in plasma using a colorimetric assay based on reduction in ferric ions (Fe^3+^) to ferrous ions (Fe^2+^) using a phenanthroline substance according to manufacturer’s instructions (Thermo Fisher EEA022). Malondialdehyde (MDA) was measured in plasma and brain homogenate by quantifying the adduct generated when MDA in the sample reacts with thiobarbituric acid (Abcam, ab118970, Lipid-Peroxidation Kit).

### Plasma analysis

Plasma aldosterone, corticosterone and 11-dehydrocorticosterone were measured by liquid chromatography tandem mass spectrometry as previously described [30]. Plasma renin was measured by ELISA (Abcam, ab193728).

### Statistical analysis

Power calculations were used to determine sample size (G*Power[45] RRID:SCR_013726) for reliable detection of differences in blood pressure as measured by telemetry. They were based on previously published differences in blood pressure between Ts65Dn mice and their wildtype littermates [35]. A sample size of 7/group was determined to be sufficient to give 80% power to detect a difference with a significance of *P*<0.05 using Cohen’s d effect size; we used 8 animals/group to allow for any complications of telemetry, but we did not have to exclude any animals from analysis. For the renal function and tissue analysis, we used 6–9 animals/group.

All data were tested for normality using the Kolmogorov–Smirnoff normality test, and the appropriate parametric or non-parametric statistical tests were used accordingly. All statistical tests used were two-tailed. Statistical comparisons were made using a Student’s t-test or Mann–Whitney U test or two-way ANOVA tests with appropriate post hoc tests (Tukey’s) for multiple groups. The asterisks in the figures indicate statistical significance: *P<0.05, **P<0.01 and ***P<0.001. All graphs were plotted with GraphPad Prism software (RRID:SCR_002798) or R ggplot (RRID:SCR_014601). Blood pressure data were analysed in two ways: first by comparison of the medians of blood pressure and heart rate during the inactive and active periods; and second by cosinor analysis which takes into account the circadian rhythm of these measures. This included the calculation of the amplitude and the midline estimating statistic of rhythm (MESOR). Amplitude is a measure of the magnitude of fluctuation in blood pressure and heart rate over the course of 24 hours. The amplitude allows us to determine the extent of drop or dipping in blood pressure which should occur during the inactive period. MESOR is the baseline or average value around which a circadian rhythm fluctuates, and unlike mean blood pressure alone, MESOR reflects the centre point of the biological rhythm. Cosinor analysis was conducted and visualised using the R packages Circacompare and Limorhyde [46,47].

## Results

### Blood pressure in *Cbr1^+/−^* mice

Mice heterozygous for *Cbr1* had increased median systolic pressure during both the active and inactive periods and increased diastolic and mean arterial pressure (MAP) during the inactive phase compared with *Cbr1^+/+^* littermate controls (Table 2). There was no difference in median heart rate between *Cbr1^+/^*^−^ and *Cbr1^+/+^* littermate controls.

**Table 2:** Median ( + IQR) blood pressure and heart rate and cosinor analysis of these parameters in mice heterozygous for *Cbr1* (*Cbr1^+/−^*) and their littermate controls (*Cbr1^+/+^*) during the inactive and active period (*n* = 8/group).

	Inactive period		Active period		MESOR		Amplitude	
	*Cbr1^+/+^*	*Cbr1^+/−^*	*Cbr1^+/+^*	*Cbr1^+/−^*	*Cbr1^+/+^*	*Cbr1^+/−^*	*Cbr1^+/+^*	*Cbr1^+/−^*
**Systolic (mmHg**)	110.2 (105.8, 113.5)	115.0 (111.9, 116.6)***	125.1 (122.5, 129.2)	127.8 (124.3, 131.1)*	116.9 (116.7, 117.1)	121.2 (121.0, 121.4)***	10.0 (9.7, 10.4)	10 (9.6, 10.3)
**Diastolic (mmHg**)	80.31 (76.47, 84.92)	83.26 (81.42, 85.19)*	94.53 (89.77, 98.67)	95.97 (94.36, 98.85)	87.6 (87.4, 87.8)	89.4 (89.2, 89.6)***	9.3 (9.2, 9.5)	9.4 (9.2, 9.6)
**MAP (mmHg**)	91.17 (86.45, 94.39)	93.96 (91.96, 95.73)**	105.9 (100.9, 108.4)	106.6 (104.7, 110.1)	96.4 (96.1, 96.6)	99.8 (99.6, 100.1)***	9.3 (8.9, 9.6)	9.5 (8.9, 9.7)
**Heart rate (bpm**)	455.9 (434.0, 479.5)	473.5 (442.3, 496.4)	521.2 (501.8, 553.9)	530.1 (512.8, 561)	492.7 (490.6, 494.8)	500.6 (498.5, 502.8)***	56.4 (53.4, 59.4)	58.0 (55.0, 61.1)

The rhythm-adjusted mean (MESOR), amplitude of each parameter and the outcome of statistical comparison of genotypes by Mann–Whitney U test are shown. * < 0.05, ** < 0.001, *** < 0.0001.

mmHg , millimetres of mercury. MAP , mean arterial pressure. bpm , beats per minute.

**Figure 1:**
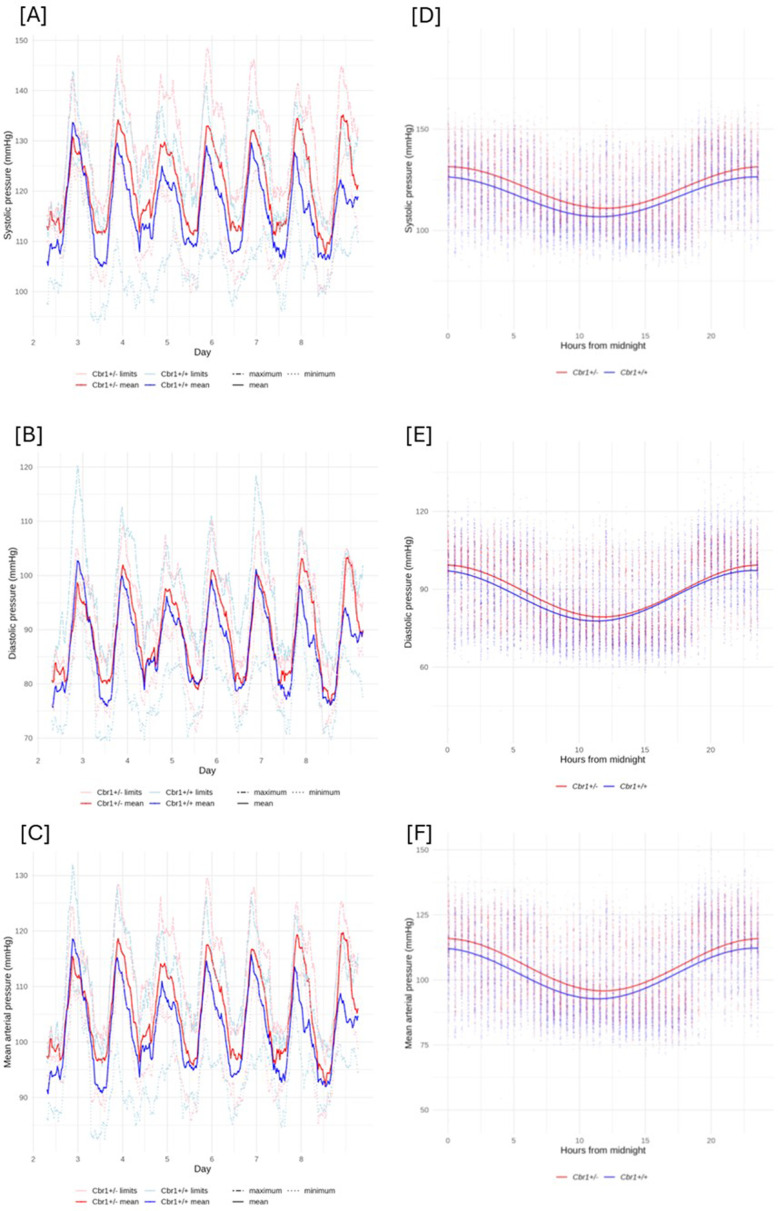
*Cbr1* deletion results in elevated blood pressure regardless of cardiac or circadian phase. The left -hand column [**A**, **B**, and **C**] shows the five5-hour rolling averages and minimum and maximum systolic, diastolic and mean arterial blood pressure of wild type mice (blue) and mice heterozygous for Cbr1 (red) (*n* = 8/group). [**D**, **E** and **F**] show the cosinoranalysis, curves fitted and spread of data points for the seven-day measurement period for systolic, diastolic and mean arterial pressure.

The blood pressure and heart rate of both *Cbr1^+/^*^−^ and *Cbr1^+/+^* littermate controls could be modelled with a cosine curve indicating a circadian rhythm, as expected. The rhythm-adjusted mean (MESOR) of systolic, diastolic and MAP was increased in *Cbr1^+/−^* compared with *Cbr1^+/+^* controls (Figure 1, Table 2). There was no difference in the amplitude between the groups for any blood pressure parameter measured. This indicates that blood pressure was increased in *Cbr1^+/−^* during both the active and inactive periods and that the magnitude of the inactive dipping was not affected by genotype (Figure 1, Table 2). The MESOR of heart rate was significantly higher in *Cbr1^+/^*^−^ compared with *Cbr1^+/+^* controls (Table 2). The amplitude did not differ between the groups for heart rate indicating *Cbr1^+/^*^−^ retained a dipping of heart rate in the inactive phase (Table 2).

### Inhibition of CBR1 in a mouse of model of DS

We hypothesised that a mouse model of DS, Ts65Dn, would have relative hypotension and that pharmacological inhibition of CBR1 would increase blood pressure.

We first confirmed that Ts65Dn mice had higher hepatic and cardiac mRNA levels and CBR1 activity (Supplementary Figure S1) compared with littermate controls. We then determined the extent of inhibition of CBR1 activity by the drug. Administration of the selective CBR1 inhibitor, hydroxy-PP-Me, reduced hepatic and brain CBR1 activity in Ts65Dn mice to equivalent to the wildtype mice but did not reduce cardiac CBR1 activity (Supplementary Figure S1).

Blood pressure was measured at baseline and during treatment with hydroxy-PP-Me. Median systolic, diastolic and MAP during both inactive period and active period were significantly lower in Ts65Dn mice compared with wildtype littermates (Table 3). Heart rate was significantly higher in the Ts65Dn mice compared with littermate controls (Table 3).

**Table 3:** Blood pressure and heart rate of Ts65Dn mice and their wildtype littermate controls (Wt) during the inactive and active period (*n* = 8/group). Data are median and interquartile range. Genotypes were compared using a Mann–Whitney U test.

	Inactive period			Active period		
	Wt	Ts65Dn	*P* value	Wt	Ts65Dn	*P* value
**Systolic (mmHg**)	114.2 (111.3, 122.1)	106.6 (101.9, 109.9)	<0.0001	124.8 (122.3, 133.3)	116.4 (110.8, 119.8)	<0.0001
**Diastolic (mmHg**)	89.95 (85.76, 100.4)	82.79 (78.95, 85.86)	<0.0001	100.6 (96.16, 109.1)	92.21 (88.94, 94.16)	<0.0001
**MAP (mmHg**)	98.31 (94.11, 107.5)	90.43 (86.55, 93.51)	<0.0001	108.5 (105.1, 117.2)	100.2 (97.13, 101.5)	<0.0001
**Heart rate (bpm**)	517.4 (496.8, 550.2)	570.7 (550.9, 600.9)	<0.001	591.7 (579, 608.9)	630.7 (618.1, 665.4)	<0.0001

mmHg , millimetres of mercury. MAP , mean arterial pressure. bpm , beats per minute.

**Figure 2:**
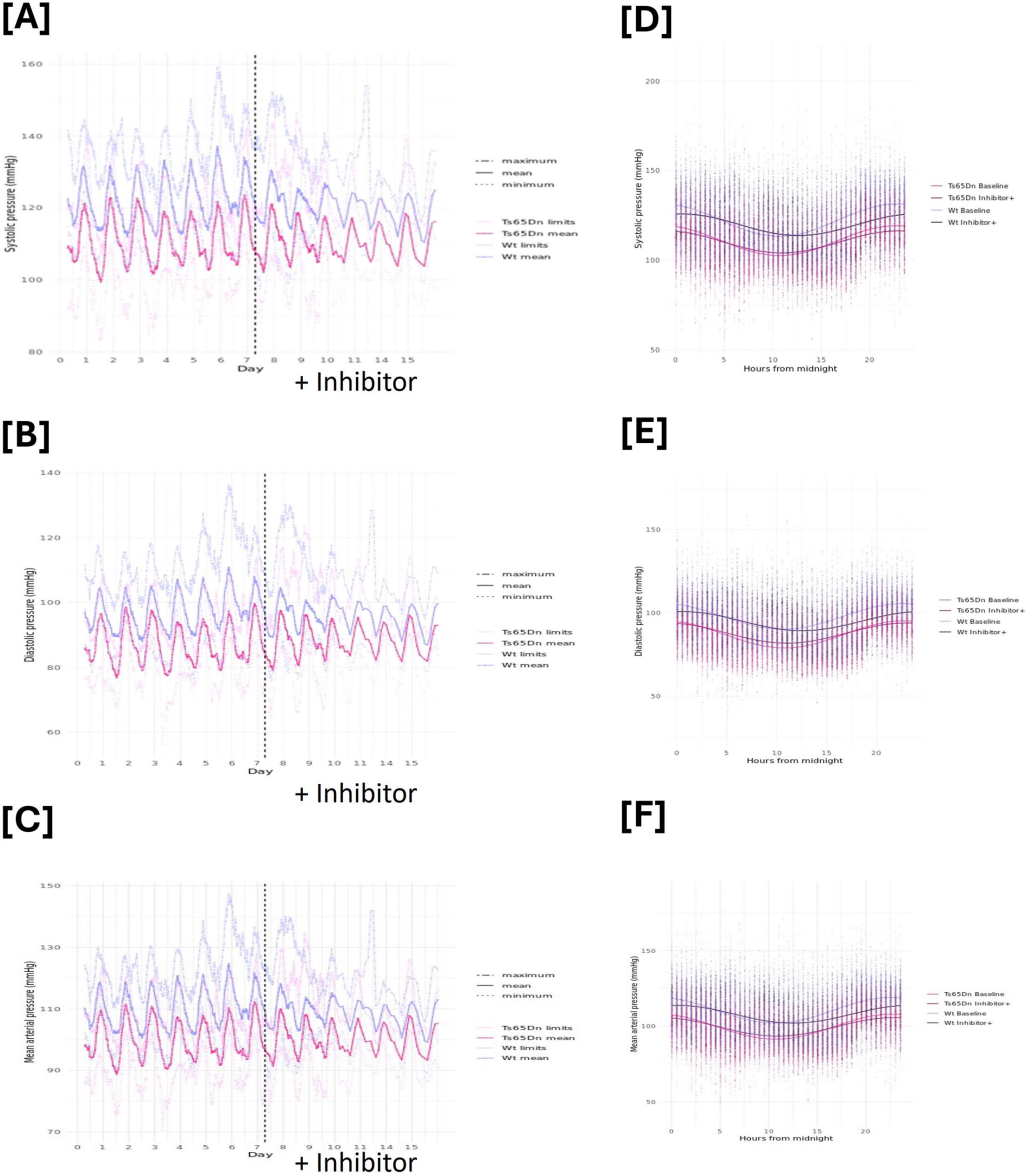
Ts65Dn mice have lower blood pressure and higher heart rate compared with Wt mice. [**A**, **B**, amnd **C**] show five5-hour rolling averages and minimum and maximum systolic, diastolic and mean arterial pressures measured by telemetry in Ts65Dn mice (pink) and their wild-type (Wt) littermate controls (purple) (*n* = 8/group) over the course of seven days of baseline measurements and then during daily treatment with CBR1 inhibitor hydroxy-PP-Me ( + inhibitor) for seven days. The dotted line denotes the start of inhibitor treatment. [**D**, **E** and **F**] showing the cosinor analysis and curves fitted for the baseline and +inhibitor periods in Ts65Dn and Wt mice.

Cosinor analysis also showed that the MESOR (the rhythm-adjusted means) of the systolic, diastolic and MAPs were significantly lower in Ts65Dn mice compared with wildtype littermates (Figure 2, Table 4). MESOR of heart rate was significantly higher in the Ts65Dn mice compared with littermate controls (Figure 2, Table 4). The amplitude of the circadian rhythm was not different between the groups for systolic pressure or heart rate. The amplitude of diastolic pressure and MAP was larger in the Ts65Dn mice compared with wildtype controls, corresponding to an increase in both active period blood pressure peak and inactive period blood pressure dip (Figure 2, Table 4).

**Table 4:** Cosinor analysis of blood pressure measured by telemetry in Ts65Dn mice and their wildtype littermate controls during the baseline period and during treatment with CBR1 inhibitor, hydroxy-PP-Me (*n* = 8/group). The rhythm-adjusted mean (MESOR), amplitude of each parameter for each genotype during baseline and treatment and the outcome of statistical comparison by two-way ANOVA and Tukey’s post hoc test.

	Baseline			Treatment			Change with treatment (*P* value)	
Systolic	Wt	Ts65Dn	Wt vs. Ts65Dn (*P* value)	Wt	Ts65Dn	Wt vs. Ts65Dn (*P* value)	Wt	Ts65Dn
MESOR (mmHg)	122.09 (121.8, 122.3)	110.9 (110.6, 111.1)	<0.001	120.0 (119.8, 120.2)	111.4 (111.3, 111.6)	<0.001	<0.001	<0.01
Amplitude (mmHg)	8.9 (8.6, 9.3)	8.8 (8.5, 9.2)	0.13	5.9 (5.6, 6.2)	6.2 (5.9, 6.5)	0.19	<0.001	<0.001
**Diastolic**								
MESOR (mmHg)	97.5 (97.2, 07.7)	87.1 (86.8, 87.3)	<0.001	95.3 (95.1, 95.4)	88.0 (87.8, 88.2)	<0.001	<0.001	<0.001
Amplitude (mmHg)	7.7 (7.4, 7.9)	8.5 (8.2, 8.8)	<0.001	5.6 (5.3, 5.9)	6.1 (5.9, 6.3)	<0.01	<0.001	<0.001
**MAP**								
MESOR (mmHg)	105.7 (105.4, 105.9)	95.0 (94.8, 95.2)	<0.001	103.5 (103.3, 103.7)	95.5 (95.3, 95.6)	<0.001	<0.001	<0.01
Amplitude (mmHg)	8.1 (7.8, 8.4)	8.6 (8.3, 9.0)	<0.01	5.7 (5.5, 6.0)	6.2 (5.9, 6.4)	<0.05	<0.001	<0.001
**Heart rate**								
MESOR (bpm)	557.5 (556.0, 558.8)	602.5 (601.1, 603.9)	<0.001	539.8 (538.5, 541.1)	564.2 (562.9, 565.5)	<0.001	<0.001	<0.001
Amplitude (bpm)	70.4 (68.4, 72.4)	69.1 (67.1, 71.1)	0.39	37.4 (35.5, 39.3)	58.1 (56. 3, 59.9)	<0.001	<0.001	<0.001

mmHg , millimetres of mercury. MAP , mean arterial pressure. bpm , beats per minutes.

Treatment with hydroxy-PP-Me significantly increased the MESOR of systolic, diastolic and MAP of Ts65Dn mice from baseline but decreased the MESOR in the wildtype mice (Figure 2, Table 4). There was a decrease in the amplitude of the rhythm in both wildtype and Ts65Dn mice corresponding to a reduction in the inactive phase dip in blood pressure, i.e. inhibition of CBR1 blunted the fall in blood pressure (Table 4). Amplitude and MESOR of heart rate were significantly reduced by treatment in both groups of mice (Table 4).

### Mechanisms altering blood pressure

To determine if the blood pressure phenotype observed in *Cbr1^+/^*^−^ was salt-sensitive*,* the animals were given a high-salt diet (3% sodium) and blood pressure was measured by telemetry for seven days. During high-salt feeding, the mean systolic, diastolic and MAP increased in both groups, but the difference between the groups remained constant (Supplementary Table 1) demonstrating that salt sensitivity was similar between the groups. We confirmed that there were no differences in renal function as measured by glomerular filtration rate between *Cbr1^+/−^* and *Cbr1^+/+^* littermate controls (Supplementary Figure S2). Renal histology determined by light microscopy of haematoxylin and eosin-stained sections was normal in both genotypes (Supplementary Figure S2). The components of the renin–angiotensin–aldosterone system were not different between the groups (Supplementary Figure S3).

We then examined vascular function in *Cbr1^+/^*^−^ and found no differences in the response of mesenteric vessels to vasoconstrictors or vasodilators to those of *Cbr1^+/+^* littermate controls (Supplementary Figure S4).

Plasma glucocorticoids (corticosterone and its inactive form 11-dehydrocorticosterone) measured by liquid chromatography tandem mass spectrometry were not different between the groups (Supplementary Figure S5).

Next, we examined known functions of CBR1 which may influence blood pressure by changing the vascular microenvironment. We explored the potential for CBR1 to affect oxidative stress, sympathetic tone and prostaglandin metabolism.

### Oxidative stress

CBR1 mediates detoxification of ROS making this a potential mechanism by which it influences blood pressure. We, therefore, looked at measures of whole-body oxidative stress (total antioxidant capacity), lipid peroxidation (TBARS assay) and urinary 8-hydroxy-2’-deoxyguanosine (8-oxo-dG), as well as brain-specific MDA. There were no differences in plasma or urinary measures of oxidative stress, but brain MDA concentrations were increased in *Cbr1^+/^*^−^ compared with *Cbr1^+/+^* littermate controls (Supplementary Figure S6).

### Sympathetic activity

**Figure 3:**
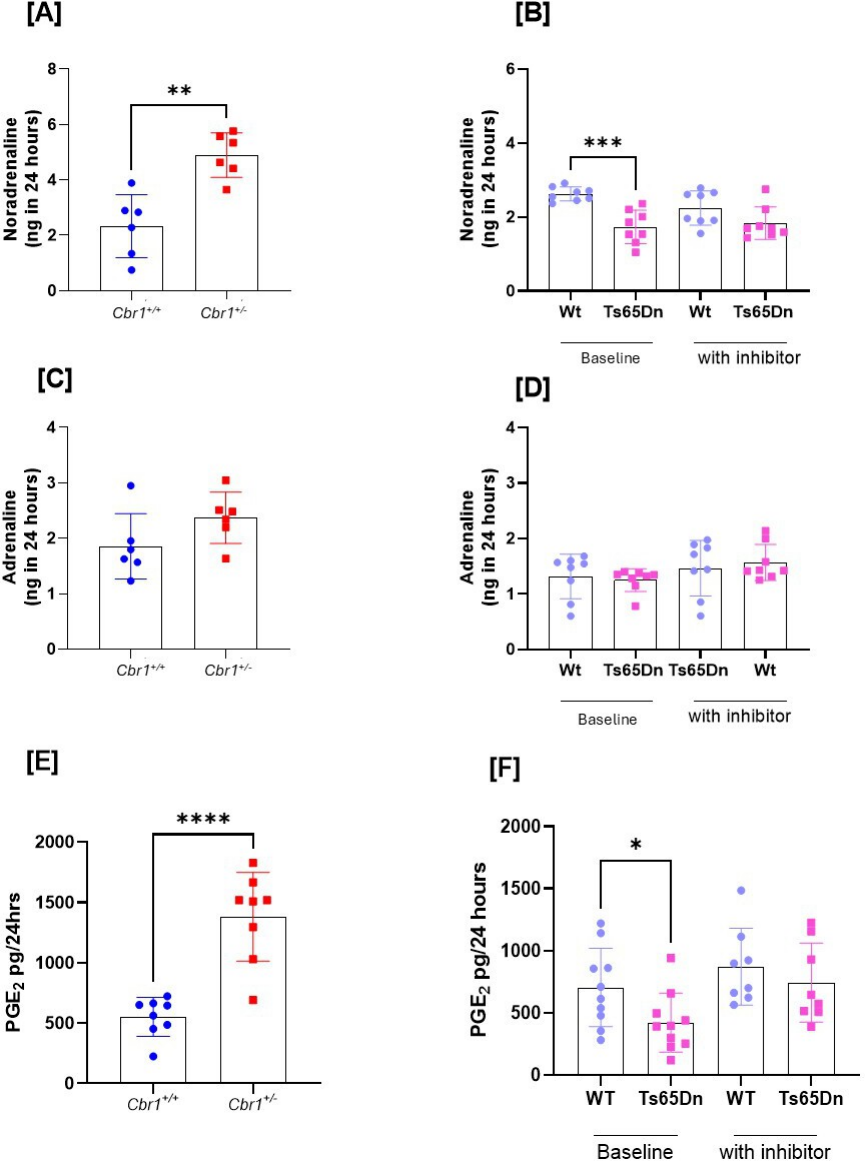
*Cbr1* deletion and inhibition results in increased sympathetic drive and prostaglandin metabolism. Urinary noradrenaline excretion in a 24-hour period was increased in mice heterozygous for *Cbr1* (*Cbr1*^*+/−*^) compared with their littermate controls (*Cbr1*^*+/+*^) [**(A**]**)** (*n* = 6/group), and the opposite was true of Ts65Dn mice who had reduced noradrenaline excretion [**(B**]**)** (*n* = 8/group). Urinary adrenaline excretion was not significantly different in *Cbr1*^*+/−*^ or Ts65Dn animals compared with wild-type controls [**(C**, **D**]**)**. [**(E**]**)** Urinary prostaglandin E_2_ excretion was increased in mice heterozygous for *Cbr1* (*Cbr1*^*+/−*^) compared with littermate controls (*Cbr1*^*+/+*^) (*n* = 8/group). PGE_2_ excretion was decreased in Ts65Dn animals compared towith controls, and this was corrected by administration of the inhibitor [**(F**]**)** (*n* = 8–11/group). Data were analysed by t-test or by ANOVA with post- hoc Tukey’s and are presented as group mean ± standard deviation (*P<0.05, **P<0.01, ***P<0.001, and ****P<0.0001).

We determined if urinary excretion of catecholamines noradrenaline and adrenaline, as a proxy for sympathetic drive, was altered in *Cbr1^+/^*^−^ compared with *Cbr1^+/+^* littermate controls. Twenty-four-hour urinary excretion of noradrenaline and adrenaline was measured in mice housed in metabolic cages. Urinary excretion of noradrenaline but not adrenaline was increased in *Cbr1^+/^*^−^ animals compared with their littermate controls (Figure 3A,C). We also showed that the mouse model of DS demonstrated decreased urinary excretion of noradrenaline but not adrenaline (Figure 3B,D). Administration of hydroxy-PP-Me normalised noradrenaline excretion in Ts65Dn animals (Figure 3).

### Prostaglandin excretion

CBR1 inactivates PGE_2_ and converts it to PGF_2α_, a mediator of blood pressure. As such, we measured excretion of the metabolites of substrate PGE_2_ in urine of mice heterozygous for *Cbr1* (*Cbr1*^+/−^) compared with their littermate controls (*Cbr1*^+/+^) and found that *Cbr1*^+/−^ mice had increased excretion indicating reduced systemic metabolism (Figure 3). The opposite was true of Ts65Dn animals compared with littermate controls, but this was normalised by administration of hydroxy-PP-Me(Figure 3).

## Discussion

In this study, we present the first demonstration of CBR1 as a novel regulator of blood pressure. Our data indicate that increased CBR1 contributes to hypotension observed in a mouse model of DS. Additionally, mice heterozygous for *Cbr1,* with a 50% reduction in enzyme activity in all tissues [30], had increased systolic, diastolic and MAP. In the absence of changes in renal function, salt sensitivity or vascular reactivity, the most plausible drivers of altered blood pressure are the observed alterations in sympathetic tone and prostanoid metabolism, inferred from urinary catecholamine and prostaglandin excretion.

It is suggested that, in DS, blunted sympathetic control is associated with exercise intolerance and low VO_2_ max [17,48] and is also implicated in sleep apnoea in these patients [11]. Others have shown a reduced catecholamine response to exercise in adults with DS[49] and a lack of vasoconstriction in response to sympathoexcitation [50]. Hypotension and exercise intolerance can have a significant impact on the quality of life for people with DS, limiting exercise, contributing to sleep disturbances and potentially accelerating the onset and progression of Alzheimer’s disease [3,5]. There are currently no specific treatments available for hypotension in DS as the pathophysiology remains unknown. Our study suggests that decreasing CBR1 activity can either genetically or pharmacologically increase sympathetic tone, particularly noradrenaline release, which can affect blood pressure control.

We cannot be sure of the mechanism by which CBR1 influences sympathetic tone; the protein is expressed throughout the brain and adrenal medulla, and its effects could be direct or indirect. We found changes in both systemic prostaglandin metabolism and brain oxidative stress relative to CBR1 activity, both of which could indirectly affect sympathetic output. PGE_2_, a substrate of CBR1 [51], is known to induce hypertension and catecholamine release when administered intracerebroventricularly to rats [52,53] and yet have the opposite effect when given systemically [54]. Reduced levels of PGE_2_ in the brain are found in the Ts1Cje rodent model of DS, and this is reversed when the copy number of the *Cbr1* gene is restored [55]. Our results are consistent with this, demonstrating that mice with reduced CBR1 activity had reduced metabolism (and hence increased excretion) of PGE_2_ metabolites. We did not identify the source of this increased PGE_2_ but given we did not see differences in plasma renin, and systemic vascular function was unaffected, we might hypothesise that the increases were localised in the brain, thereby influencing sympathetic activity or alternatively acting directly on the cerebral vasculature.

CBR1 may also affect sympathetic tone or blood pressure by alterations in oxidative stress. Oxidative stress appears to stimulate central sympathetic outflow in various models of hypertension [56], but little is reported in relation to hypotension. CBR1 is known to reduce oxidative stress centrally where it inactivates highly reactive lipids [57], and this was apparent in our work which showed increased levels of MDA in the brains of mice deficient in *Cbr1*. Serum MDA levels have consistently been found to be elevated in patients with hypertension [58] and are thought to be a marker of increased systemic oxidative stress. However, the causal direction in hypertension remains unclear [56]. Interestingly, our findings were confined to the brain and we found no evidence of a systemic increase in markers of oxidative stress in *Cbr1^+/^*^−^. This is consistent with the normal vascular and renal function we saw in these animals, and it is also likely that compensatory mechanisms come into play when *Cbr1* is lacking or that 50% of normal levels are sufficient to protect cells elsewhere. To our knowledge, our work is the first to demonstrate that a reduction or imbalance in oxidative stress may contribute to hypotension and we proffer that a perfect balance is required throughout to maintain optimal blood pressure.

CBR1 could also affect the sympathetic nervous system more directly; for example, it was recently described as the predominant pathway by which the endogenous monoamine oxidase inhibitor, isatin, is inactivated [26,59]. Increases in isatin have been associated with hypertension [60]. It is most likely that a combination of all these proposed mechanisms plays a part in the phenotype and our data suggest that there is a critical and optimal level of CBR1 activity which maintains homeostasis in the microvascular environment. Indeed, inhibition of CBR1 in wildtype animals increased the MESOR of blood pressure whilst still decreasing the amplitude of each blood pressure parameter and heart rate, suggesting that compensation is possible when CBR1 is not elevated.

Despite inhibition of CBR1 with hydroxy-PP-Me resulting in tissue-specific rather than systemic enzyme inhibition, there was still a blunting of the normal inactive phase dip in blood pressure and an increase in noradrenaline excretion in this mouse model of DS. This suggests that there is merit in pursuing CBR1 inhibition by this or other compounds [61,62] as a therapeutic intervention in patients for whom hypotension affects the quality of life. It is interesting to note that inhibition of CBR1 reduced blood pressure in the wildtype mice in whom CBR1 levels were ‘normal’ so it seems likely that a critical balance of CBR1 activity is required to maintain a normal vascular microenvironment and blood pressure; as such, partial inhibition may be an attractive therapeutic option.

Whilst we have focused on the role of *Cbr1* in DS, our work has wider implications. In the general population, there is wide variation in CBR1 expression and activity levels between the sexes and between ethnic groups [63], and our data suggest that *CBR1* may be a novel gene influencing blood pressure. Inhibitors of CBR1, particularly flavonoids, exist in many foodstuffs and food supplements [64] and are often advocated as supplements for people with metabolic disease. Pharmacological inhibitors of CBR1 are being explored for use as adjunctive therapy in chemotherapeutic regimes which include doxorubicin because CBR1 metabolises doxorubicin to cardiotoxic daunorubicin which limits its use, particularly in DS patients [21,37,65]. Our data suggest that inhibition of CBR1 should be used with caution in those with or susceptible to hypertension.

It is important to acknowledge the limitations of these studies. We used mice which were heterozygous for *Cbr1* in every tissue; therefore, we cannot ascertain which tissue or cell type is most important in the hypotensive phenotype. We acknowledge the limitations of inferences made in mice in such a complex human syndrome as DS, and the role or importance of Cbr1 in human blood pressure control may differ from that in mice. Our power calculations demonstrated that we were sufficiently powered to determine a difference in blood pressure between genotypes and with the inhibitor, and blood pressure was measured in the same animals with and without inhibitor which is a major strength of the study. However, the study may have been underpowered to detect more subtle differences in physiological changes which speak to the underlying mechanisms.

## Clinical Perspectives

- Down syndrome (DS) is the most common chromosomal disorder, affecting approximately 1 in every 800 babies born. Hypotension is common amongst children and adults with DS and often affects the quality of life. The pathophysiology of DS-associated hypotension is poorly understood.
- In this study, we identified carbonyl reductase 1 as a driver of the hypotensive phenotype in DS. Inhibition of CBR1 in a hypotensive rodent model of DS resulted in an increased blood pressure. Mice heterozygous for *Cbr1* have increased blood pressure. Mechanistic studies show that changes in sympathetic drive, oxidative stress and prostanoid metabolism underpin the effects of CBR1 on blood pressure.
- Our data suggest that CBR1 may be a potential therapeutic target in those DS patients for whom low blood pressure affects their quality of life.

## Supplementary material

Online supplementary material 1

## Data Availability

The data included in this study are available from the corresponding authors upon reasonable request.

## Abbreviations

CBR1/Cbr1: carbonyl reductase 1
DS: Down syndrome
MAP: mean arterial pressure
MDA: Malondialdehyde
MESOR: Midline Estimating Statistic of Rhythm (Rhythm-adjusted mean)
8-OHdG: 8-hydroxy-2’-deoxyguanosine
ROS: Reactive oxygen species
TBARS: Thiobarbituric acid reactive substances

## References

[1] de Graaf, G.,, Buckley F., Skotko B.G (2017). Estimation of the number of people with Down syndrome in the United States. Genet. Med..

[2] Määttä T., Määttä J., Tervo-Määttä T., Taanila A., Kaski M., Iivanainen M (2011). Healthcare and guidelines: A population-based survey of recorded medical problems and health surveillance for people with Down syndrome. J. Intellect. Dev. Disabil..

[3] Capone G.T., Chicoine B., Bulova P., Stephens M., Hart S., Crissman B. (2018). Co-occurring medical conditions in adults with Down syndrome: A systematic review toward the development of health care guidelines. Am. J. Med. Genet. A.

[4] Santoro J.D., Lee S., Mlynash M., Nguyen T., Lazzareschi D.V., Kraler L.D. (2018). Blood pressure elevation and risk of moyamoya syndrome in patients with trisomy 21. Pediatrics.

[5] Morrison R.A., McGrath A., Davidson G., Brown J.J., Murray G.D., Lever A.F (1996). Low blood pressure in Down’s syndrome, A link with Alzheimer’s disease?. Hypertension.

[6] Cilhoroz B.T., Receno C.N., Heffernan K.S., Deruisseau LR (2022). Cardiovascular physiology and pathophysiology in Down syndrome. Physiol. Res..

[7] Santoro J.D., Lee S., Mlynash M., Mayne E.W., Rafii M.S., Skotko B.G (2020). Diminished Blood Pressure Profiles in Children With Down Syndrome. Hypertension.

[8] Oviedo G.R., Carbó-Carreté M., Guerra-Balic M., Tamulevicius N., Esquius L., Guàrdia-Olmos J. (2022). Hemodynamic and cardiorespiratory responses to submaximal and maximal exercise in adults with down syndrome. Front. Physiol..

[9] Alhammad S.A., Alqahtani A.S., Alwadeai K.S., Algabbani M.F., Alhusaini A.A (2024). Walking capacity and its association with quality of life among children with down syndrome in saudi arabia. BMC Pediatr..

[10] Pitetti K.H., Baynard T., Agiovlasitis S (2013). Children and adolescents with Down syndrome, physical fitness and physical activity. J. Sport Health Sci..

[11] Santos R.A., Costa L.H., Linhares R.C., Pradella-Hallinan M., Coelho F.M.S., Oliveira G. da P (2022). Sleep disorders in Down syndrome: a systematic review. Arq. Neuropsiquiatr..

[12] Alexander M., Petri H., Ding Y., Wandel C., Khwaja O., Foskett N (2016). Morbidity and medication in a large population of individuals with Down syndrome compared to the general population. Dev. Med. Child Neurol..

[13] Baynard T., Pitetti K.H., Guerra M., Fernhall B (2004). Heart rate variability at rest and during exercise in persons with Down syndrome. Arch. Phys. Med. Rehabil..

[14] Figueroa A., Collier S.R., Baynard T., Giannopoulou I., Goulopoulou S., Fernhall B (2005). Impaired vagal modulation of heart rate in individuals with Down syndrome. Clin. Auton. Res..

[15] Iellamo F., Galante A., Legramante J.M., Lippi M.E., Condoluci C., Albertini G. (2005). Altered autonomic cardiac regulation in individuals with down syndrome. Am. J. Physiol. Heart Circ. Physiol..

[16] Fernhall B., Figueroa A., Collier S., Baynard T., Giannopoulou I., Goulopoulou S (2005). Blunted heart rate response to upright tilt in people with down syndrome. Arch. Phys. Med. Rehabil..

[17] Leti T., Guinot M., Favre-Juvin A., Bricout V.A (2015). Difference of catecholamine responses to exercise in men with trisomy 21, with or without chronotropic incompetence. Physiol. Behav..

[18] Gonzalez-Covarrubias V., Ghosh D., Lakhman S.S., Pendyala L., Blanco J.G. (2007). A functional genetic polymorphism on human carbonyl reductase 1 (CBR1 V88I) impacts on catalytic activity and NADPH binding affinity. Drug Metab. Dispos..

[19] Jiang X., Liu C., Yu T., Zhang L., Meng K., Xing Z. (2015). Genetic dissection of the down syndrome critical region. Hum. Mol. Genet..

[20] Zhang L., Meng K., Jiang X., Liu C., Pao A., Belichenko P.V. (2014). Human chromosome 21 orthologous region on mouse chromosome 17 is a major determinant of down syndrome-related developmental cognitive deficits. Hum. Mol. Genet..

[21] Forrest G.L., Gonzalez B., Tseng W., Li X., Mann J (2000). Human carbonyl reductase overexpression in the heart advances the development of doxorubicin-induced cardiotoxicity in transgenic mice. Cancer Res..

[22] Sjöstedt E., Zhong W., Fagerberg L., Karlsson M., Mitsios N., Adori C. (2020). An atlas of the protein-coding genes in the human, pig, and mouse brain. Science.

[23] Karlsson M., Zhang C., Méar L., Zhong W., Digre A., Katona B. (2021). A single-cell type transcriptomics map of human tissues. Sci. Adv..

[24] Oppermann U (2007). Carbonyl reductases: the complex relationships of mammalian carbonyl- and quinone-reducing enzymes and their role in physiology. Annu. Rev. Pharmacol. Toxicol..

[25] Rashid M.A., Lee S., Tak E., Lee J., Choi T.G., Lee J.W. (2010). Carbonyl reductase 1 protects pancreatic β-cells against oxidative stress-induced apoptosis in glucotoxicity and glucolipotoxicity. Free Radic. Biol. Med..

[26] Usami N., Kitahara K., Ishikura S., Nagano M., Sakai S., Hara A (2001). Characterization of a major form of human isatin reductase and the reduced metabolite. Eur. J. Biochem..

[27] Guo C., Wang W., Liu C., Myatt L., Sun K (2014). Induction of PGF2α synthesis by cortisol through GR dependent induction of CBR1 in human amnion fibroblasts. Endocrinology.

[28] Xiong M., Chen H., Fan Y., Jin M., Yang D., Chen Y. (2023). Tubular Elabela-APJ axis attenuates ischemia-reperfusion induced acute kidney injury and the following AKI-CKD transition by protecting renal microcirculation. Theranostics.

[29] Morgan R.A., Beck K.R., Nixon M., Homer N.Z.M., Crawford A.A., Melchers D. (2017). Carbonyl reductase 1 catalyzes 20β-reduction of glucocorticoids, modulating receptor activation and metabolic complications of obesity. Sci. Rep..

[30] Bell R.M.B., Villalobos E., Nixon M., Miguelez-Crespo A., Murphy L., Fawkes A. (2021). Carbonyl reductase 1 amplifies glucocorticoid action in adipose tissue and impairs glucose tolerance in lean mice. Mol. Metab..

[31] Olson L.E., Roper R.J., Baxter L.L., Carlson E.J., Epstein C.J., Reeves R.H (2004). Down syndrome mouse models Ts65Dn, Ts1Cje, and Ms1Cje/Ts65Dn exhibit variable severity of cerebellar phenotypes. Dev. Dyn..

[32] Herault Y., Delabar J.M., Fisher E.M.C., Tybulewicz V.L.J., Yu E., Brault V (2017). Rodent models in Down syndrome research: impact and future opportunities. Dis. Model. Mech..

[33] Harashima C., Jacobowitz D.M., Witta J., Borke R.C., Best T.K., Siarey R.J. (2006). Abnormal expression of the G-protein-activated inwardly rectifying potassium channel 2 (GIRK2) in hippocampus, frontal cortex, and substantia nigra of Ts65Dn mouse: a model of down syndrome. J. Comp. Neurol..

[34] Sarver D.C., Xu C., Velez L.M., Aja S., Jaffe A.E., Seldin M.M. (2023). Dysregulated systemic metabolism in a Down syndrome mouse model. Mol. Metab..

[35] DeRuisseau L.R., Receno C.N., Heffernan K.S., Cunningham C.M. (2019). Heart rate and blood pressure in male Ts65Dn mice: a model to investigate cardiovascular responses in down syndrome. Physiol. Rep..

[36] DeRuisseau L.R., Receno C.N., Cunningham C., Bates M.L., Goodell M., Liang C. (2023). Breathing and oxygen carrying capacity in Ts65Dn and down syndrome. Function (Oxf).

[37] Olson L.E., Bedja D., Alvey S.J., Cardounel A.J., Gabrielson K.L., Reeves R.H (2003). Protection from doxorubicin-induced cardiac toxicity in mice with a null allele of carbonyl reductase 1. Cancer Res..

[38] Tanaka M., Bateman R., Rauh D., Vaisberg E., Ramachandani S., Zhang C. (2005). An unbiased cell morphology-based screen for new, biologically active small molecules. Plos Biol..

[39] Pudlo J.S., Nassiri M.R., Kern E.R., Wotring L.L., Drach J.C., Townsend L.B (1990). Synthesis, antiproliferative, and antiviral activity of certain 4-substituted and 4,5-disubstituted 7-[(1,3-dihydroxy-2-propoxy)methyl]pyrrolo[2,3-d]pyrimidines. J. Med. Chem..

[40] Czopek A., Moorhouse R., Guyonnet L., Farrah T., Lenoir O., Owen E. (2019). A novel role for myeloid endothelin-B receptors in hypertension. Eur. Heart J..

[41] Gonzalez-Covarrubias V., Zhang J., Kalabus J.L., Relling M.V., Blanco JG (2009). Pharmacogenetics of human carbonyl reductase 1 (CBR1) in livers from black and white donors. Drug Metab. Dispos..

[42] Kassner N., Huse K., Martin H.J., Gödtel-Armbrust U., Metzger A., Meineke I. (2008). Carbonyl reductase 1 is a predominant doxorubicin reductase in the human liver. Drug Metab. Dispos..

[43] Boušová I., Skálová L., Souček P., Matoušková P (2015). The modulation of carbonyl reductase 1 by polyphenols. Drug Metab. Rev..

[44] Hunter R.W., Ivy J.R., Flatman P.W., Kenyon C.J., Craigie E., Mullins L.J. (2015). Hypertrophy in the distal convoluted tubule of an 11β-hydroxysteroid dehydrogenase type 2 knockout model. J. Am. Soc. Nephrol..

[45] Faul F., Erdfelder E., Lang A.G., Buchner A (2007). G*Power 3: A flexible statistical power analysis program for the social, behavioral, and biomedical sciences. Behav. Res. Methods.

[46] Parsons R., Garner N., Oster H., Rawashdeh O (2020). CircaCompare: a method to estimate and statistically support differences in mesor, amplitude and phase, between circadian rhythms. Bioinformatics.

[47] Singer J.M., Hughey J.J (2019). LimoRhyde: a flexible approach for differential analysis of rhythmic transcriptome data. J. Biol. Rhythms.

[48] Bricout V.A., Guinot M., Faure P., Flore P., Eberhard Y., Garnier P. (2008). Are hormonal responses to exercise in young men with Down’s syndrome related to reduced endurance performance?. J. Neuroendocrinol..

[49] Fernhall B., Baynard T., Collier S.R., Figueroa A., Goulopoulou S., Kamimori G.H. (2009). Catecholamine response to maximal exercise in persons with Down syndrome. Am. J. Cardiol..

[50] Hilgenkamp T.I.M., Wee S.O., Schroeder E.C., Baynard T., Fernhall B (2018). Peripheral blood flow regulation in response to sympathetic stimulation in individuals with down syndrome. Artery Res..

[51] Wermuth B (1981). Purification and properties of an NADPH-dependent carbonyl reductase from human brain. Relationship to prostaglandin 9-ketoreductase and xenobiotic ketone reductase. J. Biol. Chem.

[52] Kondo K., Okuno T., Saruta T., Kato E (1979). Effects of intracerebroventricular administration of prostaglandins I2, E2, F2 alpha and indomethacin on blood pressure in the rat. Prostaglandins.

[53] Feuerstein G., Adelberg S.A., Kopin I.J., Jacobowitz D.M. (1982). Hypothalamic sites for cardiovascular and sympathetic modulation by prostaglandin E2. Brain Res..

[54] Zhang Y., Guan Y., Schneider A., Brandon S., Breyer R.M., Breyer M.D. (2000). Characterization of murine vasopressor and vasodepressor prostaglandin E(2) receptors. Hypertension.

[55] Arima-Yoshida F., Raveau M., Shimohata A., Amano K., Fukushima A., Watanave M. (2020). Impairment of spatial memory accuracy improved by Cbr1 copy number resumption and GABA_B_ receptor-dependent enhancement of synaptic inhibition in down syndrome model mice. Sci. Rep..

[56] Rodrigo R., González J., Paoletto F (2011). The role of oxidative stress in the pathophysiology of hypertension. Hypertens. Res..

[57] Rashid M.A., Haque M., Akbar M (2016). Detoxification of carbonyl compounds by carbonyl reductase in neurodegeneration. Adv. Neurobiol..

[58] Zuin M., Capatti E., Borghi C., Zuliani G (2022). Serum malondialdehyde levels in hypertensive patients: a non-invasive marker of oxidative stress. a systematic review and meta-analysis. High Blood Press. Cardiovasc. Prev..

[59] Medvedev A., Buneeva O., Gnedenko O., Ershov P., Ivanov A (2018). Isatin, an endogenous nonpeptide biofactor: A review of its molecular targets, mechanisms of actions, and their biomedical implications. Biofactors.

[60] Hamaue N., Yamazaki N., Minami M., Endo T., Hirahuji M., Monma Y. (1998). Determination of isatin, an endogenous monoamine oxidase inhibitor, in urine and tissues of rats by HPLC. Gen. Pharmacol..

[61] Zhou F., Hao G., Zhang J., Zheng Y., Wu X., Hao K. (2015). Protective effect of 23-hydroxybetulinic acid on doxorubicin-induced cardiotoxicity: a correlation with the inhibition of carbonyl reductase-mediated metabolism. Br. J. Pharmacol..

[62] Jamrozik M., Piska K., Bucki A., Koczurkiewicz-Adamczyk P., Sapa M., Władyka B. (2023). In silico and in vitro assessment of carbonyl reductase 1 inhibition using ASP9521-A potent aldo-keto reductase 1C3 inhibitor with the potential to support anticancer therapy using anthracycline antibiotics. Molecules.

[63] Quiñones-Lombraña A., Ferguson D., Hageman Blair R., Kalabus J.L., Redzematovic A., Blanco J.G (2014). Interindividual variability in the cardiac expression of anthracycline reductases in donors with and without down syndrome. Pharm. Res..

[64] Hintzpeter J., Hornung J., Ebert B., Martin H.J., Maser E (2015). Curcumin is a tight-binding inhibitor of the most efficient human daunorubicin reductase--Carbonyl reductase 1. Chem. Biol. Interact..

[65] Yun M., Choi A.J., Lee Y.C., Kong M., Sung J.Y., Kim S.S. (2018). Carbonyl reductase 1 is a new target to improve the effect of radiotherapy on head and neck squamous cell carcinoma. J. Exp. Clin. Cancer Res..

