# Supplementary material for: Carbonyl reductase 1: a novel regulator of blood pressure in Down syndrome
Source: Biosci Rep. 2025 Feb 26;45(2):BSR20241636. doi: 10.1042/BSR20241636 (PMC12096947; doi:10.1042/BSR20241636)
Supplement: Online supplementary material 1 [file bsr-45-02-bsr-2024-1636-s001.doc]

**Carbonyl Reductase 1: a novel regulator of blood pressure in Down Syndrome**

Alexandra J Malbon^1, 2^, Alicja Czopek^3^, Andrew M Beekman^4^, Zoë R Goddard^4^, Aileen Boyle^5^, Jessica R Ivy^3^, Kevin Stewart^3^, Scott G Denham^6^, Joanna P Simpson^6^, Natalie Z Homer^3, 6^, Brian R Walker^7^, Neeraj Dhaun^3^, Matthew A Bailey^3^ and Ruth A Morgan^2,3,5^

^1^The Royal (Dick) School of Veterinary Studies, The University of Edinburgh, Easter Bush Campus, EH25 9RG

^2^The Roslin Institute, The University of Edinburgh, Easter Bush Campus, EH25 9RG

^3^Centre for Cardiovascular Science, The Queen’s Medical Research Institute, The University of Edinburgh, Edinburgh, EH16 4TJ

^4^School of Pharmacy, University of East Anglia, Norwich Research Park, Norwich, Norfolk, NR4 7TJ

^5^Department of Animal and Veterinary Sciences, Scotland’s Rural College, Roslin Institute Building, Easter Bush Campus, EH25 9RG

^6^Mass Spectrometry Core, Edinburgh Clinical Research Facility, Queen’s Medical Research Institute, University of Edinburgh, EH16 4TJ

^7^Clinical and Translational Research Institute, Newcastle University, Newcastle upon Tyne, United Kingdom

**Corresponding Author:**

Alexandra Malbon, The Royal (Dick) School of Veterinary Studies, The University of Edinburgh, Easter Bush Campus, EH25 9RG

Tel: +447739079919

Email: [Alexandra.malbon@ed.ac.uk](mailto:Alexandra.malbon@ed.ac.uk)

Total word count: 6845

**Short Title:** Carbonyl reductase 1 and blood pressure

**Supplementary Tables**

**Table S1: The effect of high salt diet on systolic, diastolic, mean arterial pressu**re and heart rate.

|  | **Baseline- Chow Diet** | | | **High salt** | | | **Change with salt** | |
| --- | --- | --- | --- | --- | --- | --- | --- | --- |
| **SYSTOLIC** | ***Cbr1^+/+^*** | ***Cbr1^+/-^*** | ***Cbr1^+/+^ vs Cbr1^+/-^*** | ***Cbr1^+/+^*** | ***Cbr1^+/-^*** | ***Cbr1^+/+^ vs Cbr1^+/-^*** | ***Cbr1^+/+^*** | ***Cbr1^+/-^*** |
| MESOR (mmHg) | 116.3 | 120.8 | <0.0001 | 117.7 | 124 | <0.0001 | <0.0001 | <0.0001 |
| Amplitude (mmHg) | 9.47 | 9.67 | 0.47 | 12.51 | 13.69 | <0.0001 | <0.0001 | <0.0001 |
| **DIASTOLIC** |  | | | | | | | |
| MESOR (mmHg) | 86.51 | 89.28 | <0.0001 | 88.96 | 92.09 | <0.0001 | <0.0001 | <0.0001 |
| Amplitude (mmHg) | 9.32 | 9.42 | 0.67 | 11.19 | 12.37 | <0.0001 | <0.0001 | <0.0001 |
| **MAP** |  | | | | | | | |
| MESOR (mmHg) | 96.44 | 99.80 | <0.0001 | 98.54 | 102.73 | <0.0001 | <0.0001 | <0.0001 |
| Amplitude (mmHg) | 9.36 | 9.50 | 0.58 | 11.63 | 12.81 | <0.0001 | <0.0001 | <0.0001 |
| **HEART RATE** |  | | | | | | | |
| MESOR (bpm) | 516.64 | 521.04 | <0.0001 | 530.55 | 538.17 | <0.0001 | <0.0001 | <0.0001 |
| Amplitude (bpm) | 61.96 | 64.00 | 0.26 | 69.22 | 72.35 | 0.05 | <0.0001 | <0.0001 |

**Supplementary Figures:**

**[D]**

**Fig. S1: CBR1 activity (A, B, C) and mRNA transcript levels of *Cbr1* (D) are higher in hepatic and cardiac tissue of Ts65Dn mice as compared to wild type mice. CBR1 activity is reduced by hydroxy-PP-Me in hepatic and brain tissue but not cardiac tissue.** mRNA transcript levels are expressed relative to housekeeping gene 18s. *(*P < 0.05, **P < 0.01, ***P < 0.001, and ****P < 0.0001)*

**[B]**

**[A]**

| 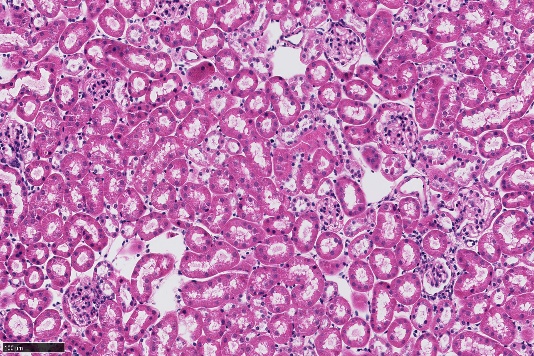 | 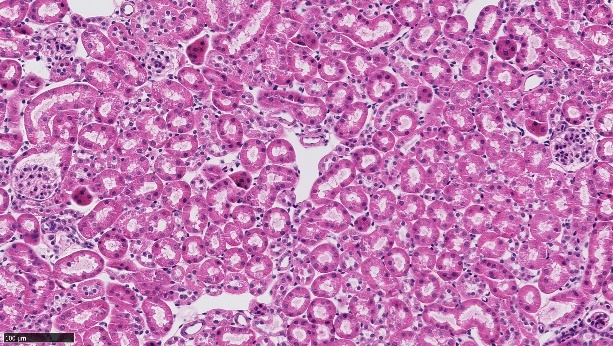 |
| --- | --- |
| ***Cbr1^+/+^*** | ***Cbr1^+/-^*** |

**Fig. S2: *Cbr1* deletion does not affect renal structure or function.** Glomerular filtration rate (GFR) [A] and representative photomicrographs of H&E stained perfusion fixed kidneys [B]. Neither GFR nor histology were different between controls (*Cbr1^+/+^)* and mice heterozygous for *Cbr1* (*Cbr1^+/-^)* (n=4-6/group). Data are mean +/- SD. Scale bar = 100µm.

**Fig. S3: *Cbr1* deletion did not affect circulating concentrations of aldosterone [A] or renin [B]** in male mice heterozygous for *Cbr1* and wild-type littermate controls (n= 6-9/group) on a normal (0.3% Na) or high-salt (3% Na) diet. Data are presented as group mean ± SD.

**
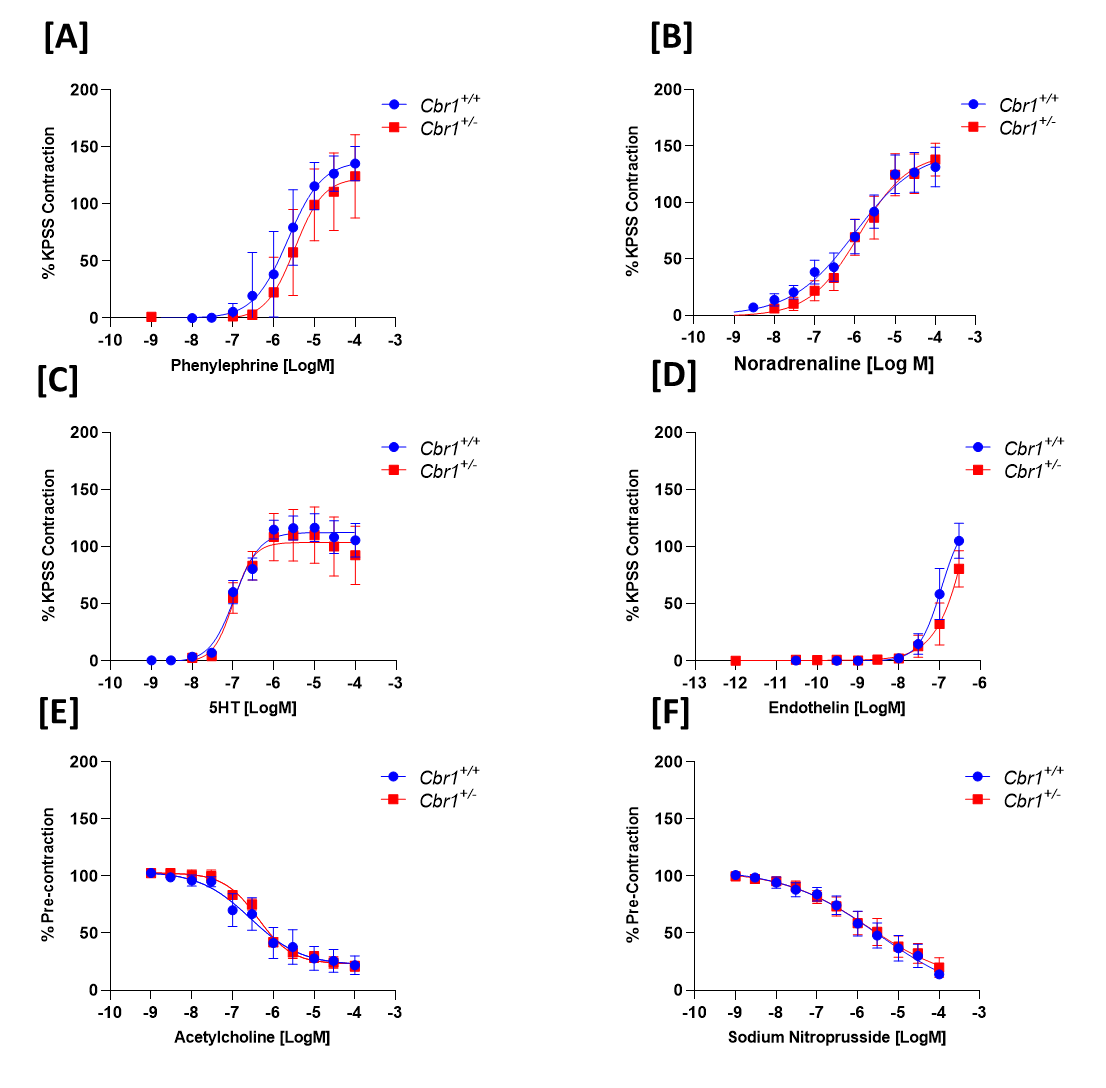
**

**Fig. S4: *Cbr1* deletion does not impact the function of mesenteric vessels.** The responses of intact mesenteric vessels to vasoconstrictors phenylephrine [A], noradrenaline [B], 5-hydroxytryptamine (serotonin, 5-HT) [C] and endothelin [D] expressed as a percentage of contraction induced by high potassium saline (KPSS) were not different between genotypes. The response to vasodilators acetylcholine [E] and sodium nitroprusside [F] expressed as a percentage of the phenylephrine-induced tension remaining from pre-constriction were not different between the groups; n=5-6/group. Log(dose) curves were produced for the calculation of logEC50 and %Emax for vasoconstrictors, and logIC50 and %Relax for vasodilators. Data are presented as group mean ± SD.

**[A]**

**[B]**

**Fig. S5: *Cbr1* deletion did not affect plasma concentrations of corticosterone or 11-dehydrocorticosterone measured by LC-MS/MS.**


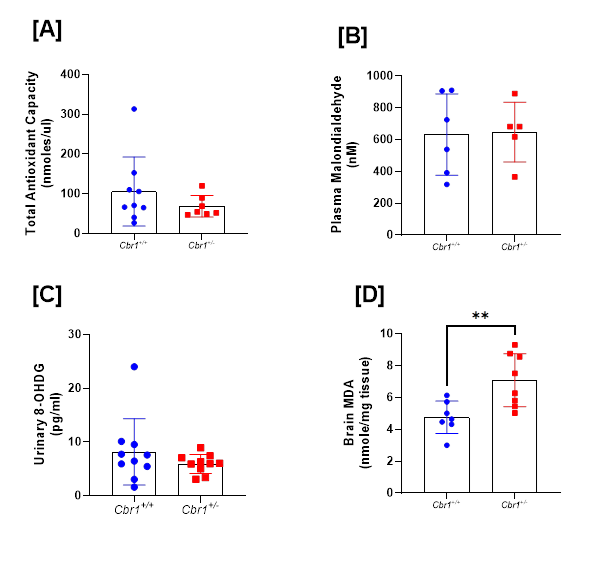


**Fig. S6: Oxidative stress markers were increased in the brains of mice heterozygous for Cbr1.** Markers of oxidative stress, plasma malondialdehyde (MDA, TBARS assay) [A] and total antioxidant capacity [B] and urinary 8-hydroxy-2’-deoxyguanosine (8-oxodG) [C] were not different between mice heterozygous for Cbr1 (Cbr1^+/-^) and their control littermates (Cbr1^+/+^) (n=8-11/group), levels of MDA in the brains of mice heterozygous for Cbr1 were increased compared with their littermate controls (n=8-11/group). Data are mean ± standard deviation (*P < 0.05, **P < 0.01, ***P < 0.001, and ****P < 0.0001)
